# Soil radon measurements as a potential tracer of tectonic and volcanic activity

**DOI:** 10.1038/srep24581

**Published:** 2016-04-15

**Authors:** Marco Neri, Elisabetta Ferrera, Salvatore Giammanco, Gilda Currenti, Rosolino Cirrincione, Giuseppe Patanè, Vittorio Zanon

**Affiliations:** 1 Istituto Nazionale di Geofisica e Vulcanologia, Osservatorio Etneo, Sezione di Catania, Piazza Roma, 2-95123 Catania, Italy; 2 Dipartimento di Scienze Biologiche, Geologiche e Ambientali – Università di Catania. C.so Italia 57, 95129 Catania, Italy; 3 Centro de Vulcanologia e Avaliação de Riscos Geológicos, Universidade dos Açores, Ponta Delgada, Portugal; 4 Institut de Physique du Globe de Paris, 1, rue Jussieu - 75238 Paris cedex 05, France

## Abstract

In Earth Sciences there is a growing interest in studies concerning soil-radon activity, due to its potential as a tracer of numerous natural phenomena. Our work marks an advance in the comprehension of the interplay between tectonic activity, volcanic eruptions and gas release through faults. Soil-radon measurements, acquired on Mt. Etna volcano in 2009–2011, were analyzed. Our radon probe is sensitive to changes in both volcanic and seismic activity. Radon data were reviewed in light of the meteorological parameters. Soil samples were analyzed to characterize their uranium content. All data have been summarized in a physical model which identifies the radon sources, highlights the mechanism of radon transport and envisages how such a mechanism may change as a consequence of seismicity and volcanic events. In the NE of Etna, radon is released mainly from a depth of <1400 m, with an ascent speed of >50 m/day. Three periods of anomalous gas release were found (February 2010, January and February 2011). The trigger of the first anomaly was tectonic, while the second and third had a volcanic origin. These results mark a significant step towards a better understanding of the endogenous mechanisms that cause changes in soil-radon emission at active volcanoes.

Radon is a radioactive noble gas naturally released by all rocks. It is used by the scientific community as a tracer of natural phenomena related to outgassing from the soil along faults, fractures and crustal discontinuities^1^,^2^,^3^,^4^,^5^,^6^,^7^,^8^. Recently, radon has also been used on active volcanoes such as Etna, both as a precursor of volcanic phenomena^9^,^10^,^11^,^12^,^13^ and as a key-parameter in the study of the dynamics of faults^14^,^15^,^16^,^17^,^18^,^19^, including those that are buried by recent lavas or tephra^20^,^21^.

In this paper, we analyze soil-radon measurements continuously recorded by a probe located on the upper northeastern flank of Mt. Etna (ERN4 in Fig. 1), near a seismogenic fault^22^,^23^,^24^, in the timeframe from 19 November 2009 to 13 April 2011. The monitoring site is also close to the NE Rift, a first-order volcano-tectonic structure of Mt. Etna, where frequent lateral volcanic eruptions occur^25^. Due to its strategic location, our probe is sensitive to radon emissions related to changes both in volcanic and seismic activity.

The collected dataset (radon activity, down-hole temperature and barometric pressure) was analyzed and compared with the main meteorological parameters recorded at the surface. Furthermore, we included seismic and volcanological data recorded on Mt. Etna by the monitoring network of the Istituto Nazionale di Geofisica e Vulcanologia – Osservatorio Etneo (INGV-OE). In order to study the potential radon emission (i.e., the fraction of radon atoms released into a rock or soil pore space from a radium-bearing grain) of the rocks at depth beneath the ERN4 station, six rock samples have been collected from lava outcrops located both inside the study area and around it. We also collected four samples of sedimentary rocks that presumably represent the pre-volcanic basement of Mt. Etna (Table 1), according to the available stratigraphic data^26^,^27^. All samples were analyzed for their main geochemical features and mostly for their uranium content.

Lastly, we used all of the acquired data to produce a physical model of the study area, in order to identify the possible sources of radon, to study the mechanism of radon transport in the upper crust of Mt. Etna and to envisage how such mechanism may change in response to variations in seismic and volcanic activity.

Mt. Etna is the most active volcano in Europe. It stands about 3,330 meters above the sea level (asl) and is located on the north-eastern coast of Sicily, Italy (Fig. 1b). The volcanic edifice rests on a sedimentary basement consisting of clay, quartzarenite and crystalline units in the north and west sectors, thrusted in the Eocene-Quaternary age, and of clayey-silt bearing Miocene – lower Pleistocene units in the eastern and southern sectors. The latter were tectonically moved to their present position during lower Pleistocene and are presumably resting on the Hyblaean limestones sequences^26^,^27^.

The flanks of Mt. Etna are crossed by three main networks of eruptive fissures, known as volcanic rifts^25^ (Fig. 1b). In the northern sector, the NE Rift is 1–2 km wide and over 7 km long. It strikes N and NE, reaching its lowest elevation at about 1500 m asl. The S Rift extends along the southern flank, spreading like a fan at low altitudes. The W Rift develops from the summit of the volcano towards the west. The area of investigation lies near the NE Rift. This rift is bounded to the east by a N60°E seismogenic fault, producing a sub-vertical ~200-meters-high step. This fault is located along the western portion of the Pernicana Fault System^28^, a major left-transtensional seismogenic faults system that underwent frequent historical seismic activity^23^ and that is highly important for understanding the tectonic setting of the volcano^24^.

## Results

**Figure 1:**
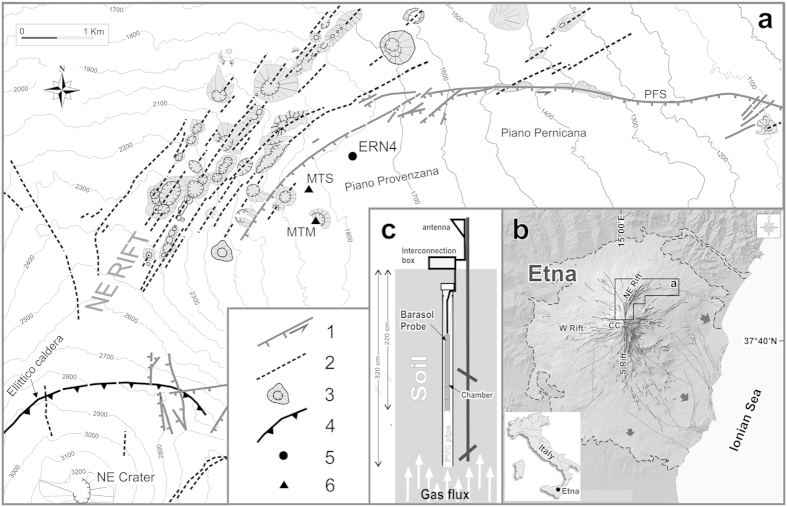
The area of investigation. Volcano-tectonic scheme of the NE sector of Mt. Etna (**a**). The radon probe (ERN4) is located a few hundred meters East of the NE Rift, and close to the western segment of the Pernicana Fault System (PFS); 1. Faults (bars on downthrown side; arrows indicate the direction of lateral movement) 2. Eruptive fissures, 3. Pyroclastic cones 4. Caldera rim (dashed if inferred), 5. Location of the ERN4 probe. 6. Location of the Meteomont (MTM) and Meteo Sicilia (MTS) weather stations, used to record meteorological parameters. (**b**) Sketch-map of Mt. Etna (outcropping volcanics are bounded by the dashed line). The black arrows indicate the direction of movement of the sectors affected by flank instability. The black lines mark eruptive fissures, which are concentrated in three rift zones (W Rift, S Rift, NE Rift). The grey lines mark outcropping faults. (**c**) Diagram (not to scale) of the set-up of the ERN4 radon probe. A PVC pipe (diameter ϕ = 10 cm), open at the bottom, is placed inside a 320 cm deep hole. A punched cap is set on the tube edge to allow ventilation. The accumulation chamber is placed at a depth of ~220 cm. Inside the interconnection box, a serial port for data downloading is housed. This figure was generated using CorelDRAW graphic suite X4 software (http://www.corel.com/it/). The topography is based on a DEM owned by INGV.

**Figure 2:**
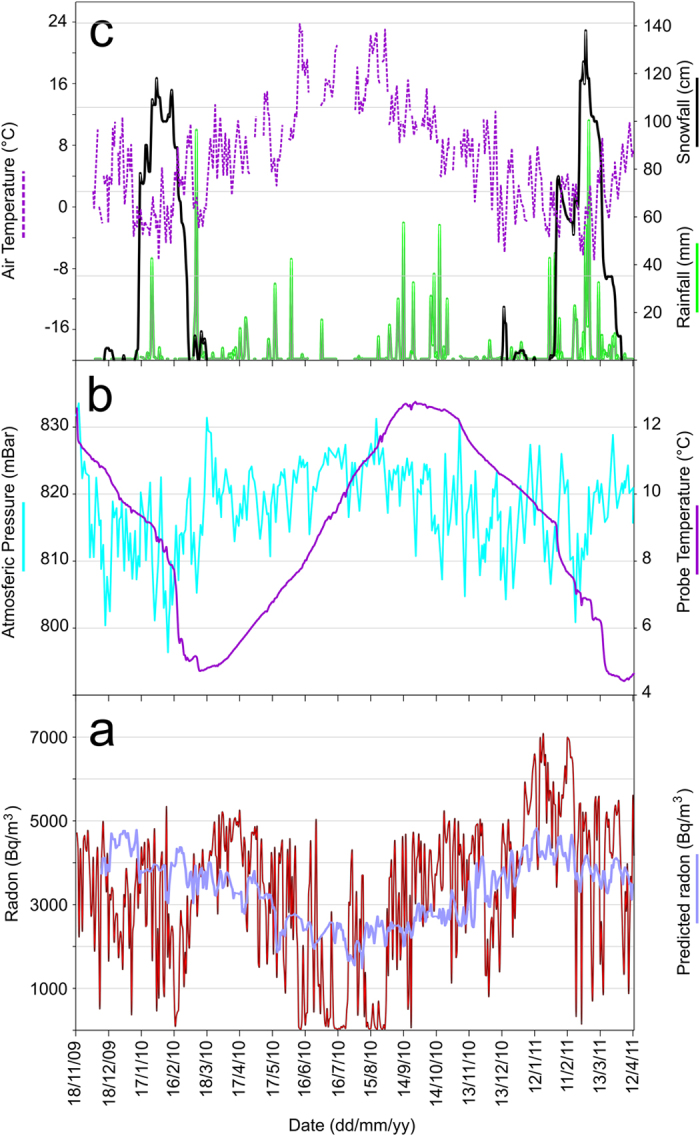
Temporal patterns of daily averages of: (**a**) soil radon activity collected in the survey site, together with the radon signal (lilac line) predicted from the linear regression model based on the values of air temperature, barometric pressure, probe temperature, snowfall (see Supplementary Table S1–4), according to the procedure described in the text; (**b**) temperature (purple line) and atmospheric pressure (blue) detected by the ERN4 probe at the hole bottom; (**c**) air temperature (dotted line), rainfall (green line) and thickness of the snow cover (black) measured by Meteo Sicilia and Meteomont weather stations.

The ERN4 radon probe (model Barasol, Algade, France) was installed in November 2009 at Piano Provenzana (1800 m asl; Fig. 1). The probe is placed at 2.2 m depth, inside a PVC pipe (for details see diagram in Fig. 1c). In addition to radon, the probe also measures atmospheric pressure and temperature (Fig. 2 and Supplementary Table S1).

**Figure 3:**
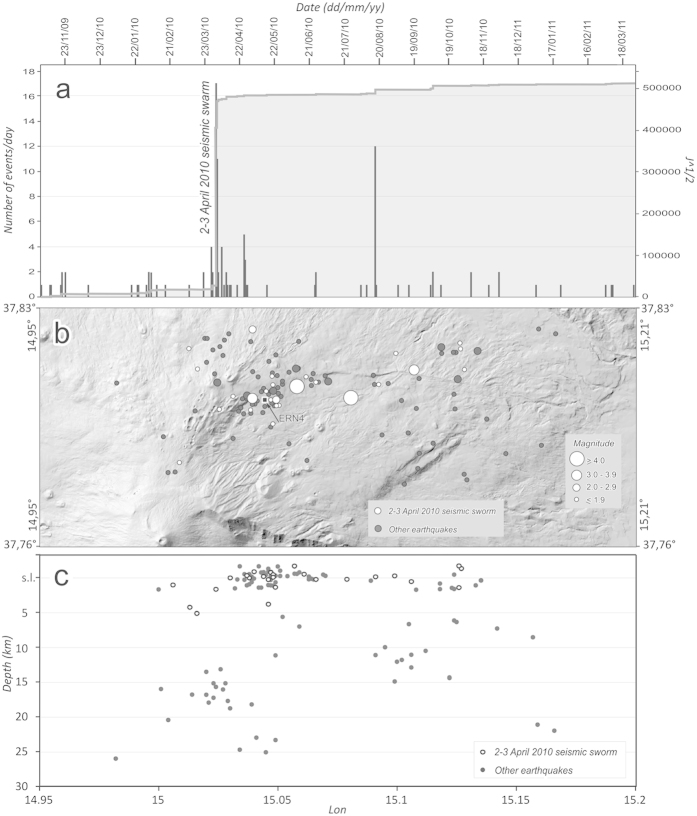
Seismicity of the study area from 2 November 2009 to 13 April 2011. (**a**) Cumulated strain release and daily frequency of earthquakes. (**b**) Epicenters distribution projected onto a Digital Elevation Model. (**c**) Distribution of hypocenters at depth. The events of the seismic swarm of the 2–3 April 2010 are represented by white circles. This figure was generated using CorelDRAW graphic suite X4 software (http://www.corel.com/it/). The topography is based on a DEM owned by INGV.

A seismic swarm affected the western portion of the Pernicana Fault System on 2–3 April 2010 (the strain release, the epicenters and hypocenters are represented in Fig. 3a–c, respectively). The swarm consisted of four very shallow (0–2 km deep) events of magnitude Ml > 3.5, which caused significant ground deformation and damage to nearby buildings^24^,^29^,^30^.

In recent years, Etna has erupted intermittently from a summit vent located on the lower eastern flank of the Southeast Crater^31^. Since January 2011, this vent has produced more than fifty paroxysmal eruptive episodes that have built up a huge pyroclastic cone, named “New Southeast Crater”^32^,^33^,^34^. The first three paroxysms in the above-mentioned sequence occurred during the final part of the period here analyzed, namely on 11–12 January, 18 February and 10 April 2011, respectively.

The first paroxysmal episode was preceded by Strombolian activity that began on 2 January 2011. Ten days later, in the evening of 12 January, the intensity of eruptive activity gradually increased, reaching its climax at 21:50 GMT, with the formation of a sustained pyroclastic column which reached a height of over 4000 m above the top of Etna. Lava fountaining reached a height of 500 m from the vent, while lava flows expanded eastward for a length of 4.3 km. The eruption ended in the early hours of 13 January^31^.

The second eruptive paroxysm started at 03:30 GMT on 18 February 2011 and lasted ~11 hours. A new lava flow expanded into the Valle del Bove, following approximately the same path as before.

In the morning of 8 April 2011, a third paroxysmal eruption began from the New Southeast Crater, initially preceded by moderate Strombolian activity that progressively increased in intensity on 9 April. The climax was reached at 13.30 UTC on 10 April and lasted about 40 minutes, ending after ~1 hour.

### Statistical analysis of the radon time series

The concentration of radon in soil gas can be influenced by many environmental factors^35^,^36^,^37^,^38^. We have, therefore, investigated the influences of the most important among these factors (namely, air temperature, sensor temperature at the hole bottom, barometric pressure, rainfall, snowfall) on the radon signal acquired to possibly filter them out. As a first step, the correlation matrix among radon and the considered meteorological parameters has been calculated. Before doing this, we filled the gaps in the air temperature values (due to malfunctioning of the temperature sensor) by applying spline functions to the original data on limited time windows starting one month before each gap and ending one month later. These treated data were then used in the following analysis.

Supplementary Table S2 shows no significant correlation (i.e., no correlation with R values >0.5) among the daily averages of radon activity values and the local meteorological parameters, thus suggesting that the latter had no strong influence on soil radon emissions. However, weak correlations (0.3 < R < 0.5 in absolute values) can be found between radon activity and air temperature and between radon activity and snowfall.

Notwithstanding the general low correlation among all parameters, we tested another independent method to detect any possible influence of the main environmental factors on the radon signal. A regression analysis, based on a linear predictive model, was performed to estimate the contribution of main meteorological parameters (set as independent variables) to the radon signal (set as dependent variable). The statistical significance of the independent variables was verified with the non-parametric Wilcoxon test^39^ (Supplementary Table S3). As a first step in this analysis, we have identified the relevant parameters that explain the variability of radon activity in our site and used the “best subset search” procedure described by Garside^40^. This procedure is based on the fitting of any possible regression equation involving the independent variables to the original radon time series, with selection of the cases that best match a chosen criterion (in our case, based on the R^2^ regression coefficient). We tested this procedure in progressive steps, calculating the R^2^ coefficient between regression equation and original radon data obtained using at first only one independent variable, and then adding a new single variable at time. When the addition of new variables to the regression equation did not improve the value of R^2^, the procedure ended and that R^2^ value was chosen as representative of the best regression. In our case, the best regression equation was that involving four out of five of the independent variables (i.e., equation four in Supplementary Table S4, with air temperature, barometric pressure, probe temperature and snowfall).

The equation representing the best linear regression model for our data is:


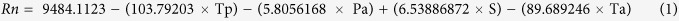


**Figure 4:**
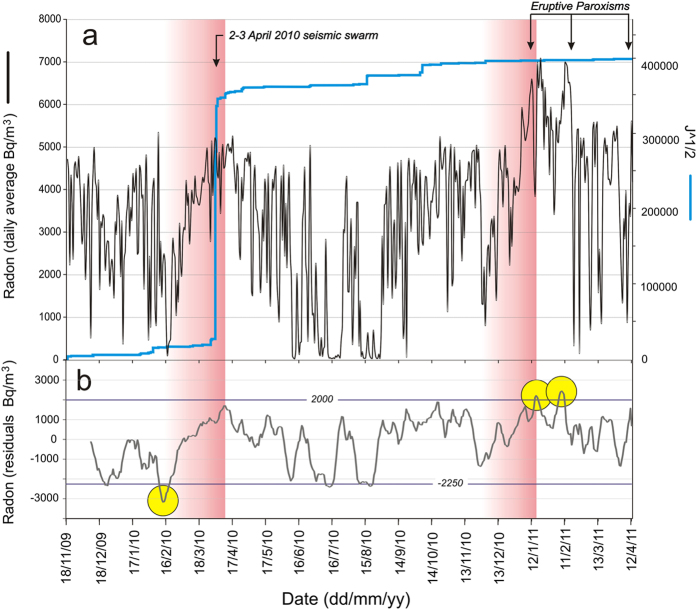
(**a**) Comparison between different signals: daily averages of radon activity, strain release associated with seismic events and paroxysmal eruptions of Mt. Etna (black arrows) occurring during the studied period. The vertical pink bands indicate the periods of steady and fairly regular increase in radon activity which lasted ~7 weeks and which preceded the seismic and eruptive events. (**b**) temporal pattern of residual radon values obtained from the linear regression analysis, with the indication of the anomaly thresholds (horizontal lines), obtained from the normal-probability plot (see Fig. 5). Yellow circles indicate main anomalous radon residuals.

We therefore used the above equation to estimate the radon changes influenced by the considered meteorological parameters (lilac line in Fig. 2a), and subtracted them from the original radon time series. This filtering procedure has provided residual radon values that could at least be partially attributed to an endogenous source. The temporal evolution of the resulting residuals is shown in Fig. 4.

In order to reveal the presence of different statistical data populations in the residual radon time series, which would help discriminating between the background and anomalous values, we have used a probability analysis based on normal distribution plots^41^. In our case, we have plotted the relative quantile of a standardized normal distribution of residual radon data (on the y-axis) versus their corresponding values (on the x-axis). If the points in the graph are aligned along a straight line with positive slope, then the observed data roughly follow a normal-law distribution and they can be considered as a single population. Conversely, if points form a broken line with different slopes for each segment, then they are indicative of separate data populations and values corresponding to slope changes for each population.

**Figure 5:**
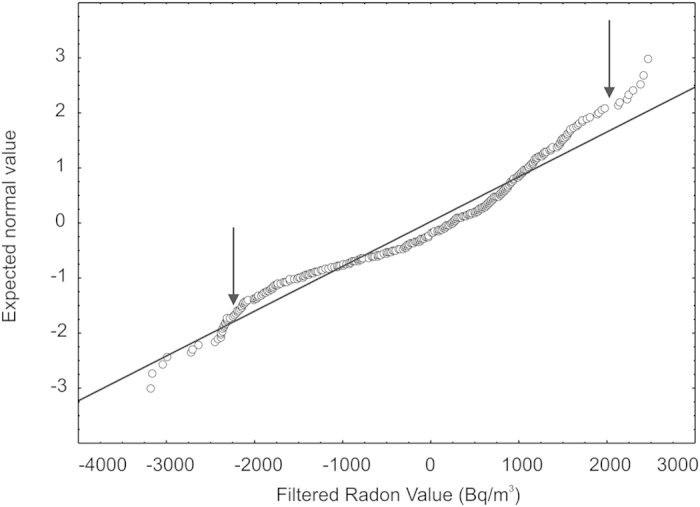
Normal probability plot of the residual values of radon activity, after filtering using the linear regression analysis described in the text. Arrows indicate the threshold values that delimit anomalies (i.e. below −2250 Bq/m^3^ and above +2000 Bq/m^3^).

In our case, the distribution of radon values does not follow a straight line (Fig. 5), thus indicative of different populations in their temporal pattern. This may be related to different conditions of radon transport through the soil. Three main slope changes are visible in the plot, identifying at least four different populations (respectively, with values <−2250 Bq/m^3^; between −2250 and +300 Bq/m^3^; between +300 and +2000 Bq/m^3^ and >+2000 Bq/m^3^). The two intermediate populations, that entail the largest number of values, are likely the expression of radon emissions related with the normal (background) soil gas variations, probably due to radon background noise and/or to other minor environmental factors (e.g., Earth tides, wind speed, soil moisture) which were not available. For the purposes of our study, therefore, we have focused only on the populations at the edges of the plot, marking residual values lower than −2250 Bq/m^3^ and higher than +2000 Bq/m^3^. These two values are the threshold values of the populations with, respectively, the lowest and the highest residual values. All these radon residuals must be considered anomalous.

Figure 4b shows that anomalous radon residuals occurred in six periods, i.e. on 25–27 December 2009, 12–20 February 2010, 12–17 July 2010, 19–22 August 2010, 16–18 January 2011 and on 7–11 February 2011. However, the most pronounced anomalies were those observed during the second, the fifth and the sixth periods. Therefore, these three anomalies will be treated in the following sections, as they are the only meaningful anomalies and the only ones that may be correlated to seismic and volcanic events at Mt. Etna.

These three anomalies in residual radon values actually occurred before the major seismic events and soon after the first paroxysmal eruption observed in the study period. The onset of the first radon anomaly (12 February 2010) preceded by 49 days the largest release of seismic energy in the area (on 2–3 April 2010). This anomaly was characterized by negative residual values, and actually the radon signal first showed a sharp drop until reaching its minimum on 15 February 2010 (−3181 Bq/m^3^, see yellow circle in Fig. 4b) and then a steady and regular increase that reached its highest values on March 29 (+1708 Bq/m^3^), thus immediately before the shocks (see left pink vertical band in Fig. 4).

Both the second and the third radon anomalies appeared in between the first two paroxysmal eruptions of Mt. Etna (on 12–13 January and 18 February 2011, respectively). As for the first anomaly and unlike the other periods, residual radon values immediately before the second anomaly showed a steady and fairly regular increase, starting from a relative minimum on 27 November 2010 (−1371 Bq/m^3^ see right pink vertical band in Fig. 4). Residual values reached their peak on 17 January 2011 and then again on 9 February 2011, to then decrease markedly and fluctuate around lower and more stable levels (in the range −1200 to +1600 Bq/m^3^) until the end of the study period.

### Model of radon transport to the surface: source zones and gas carrier velocity

In order to understand how the observed radon anomalies were produced, we have studied the mechanisms through which radon is generated in the rocks at depth and the way it is carried to the surface. To achieve this goal, we studied the stratigraphic succession below ERN4 station, calculated the potential radon emission from these rocks and modeled the transport of the gas in the sub-surface.

**Figure 6:**
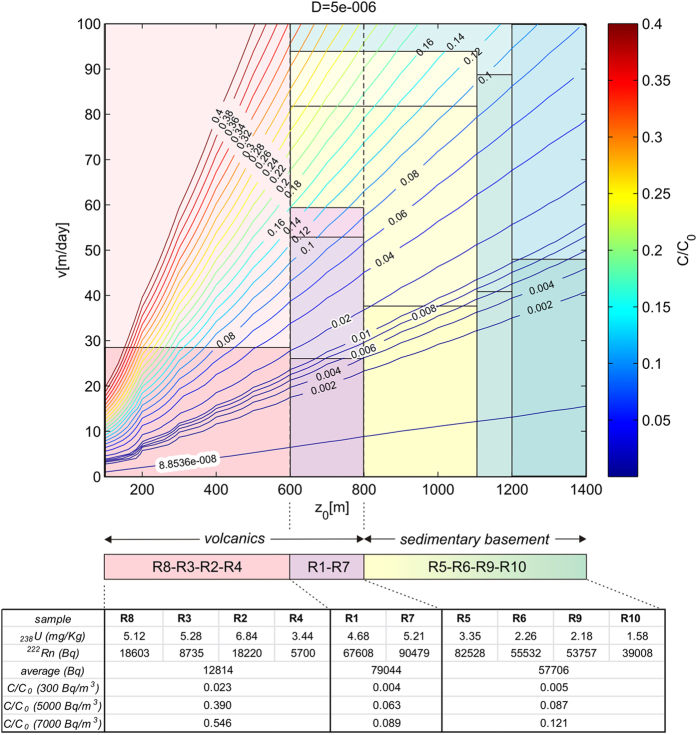
Radon concentration ratios *C/C*_*0*_ for different values of depth (z_0_) and gas carrier velocity (v). The colored boxes in the diagram indicate ranges of gas carrier velocity for different intervals of depth. R1–10 = Rock samples here analyzed. The location map of the rocks sampling points is shown in Supplementary Map S6 on line. See text for details.

The supposed stratigraphic succession underlying the ERN4 radon probe is made up, from top to bottom, of volcanic rocks (thickness ~800 m) and sedimentary rocks belonging to the basement of Mt. Etna (depth >800 m)^42^,^43^. The upper (0–600 m) volcanic rock units are characterized by low potential emissivity (average 12,814 Bq; see Fig. 6 and Table 1, S5), while it is higher in the lower volcanic units (600–800 m) (average 79,044 Bq). Also the rock of the sedimentary basement (>800 m) has a potential for radon emissivity (average 57,706 Bq).

Radon is a dense gas and therefore requires a transporter to move across the crust. Its movement over long-distances in the sub-surface is governed by a combination of molecular diffusion, described by Fick’s Law, and advection by means of a carrier gas flow, described by Darcy’s Law^44^. In the case of our site on Mt. Etna, it is likely that the latter prevails, given the relatively high soil-CO_2_ emissions of volcanic origin that are normally measured on the flanks of the volcano^11^,^44^,^45^,^46^. Because of the short life-time of radon, its transport towards the ground surface requires a relatively fast-moving advective gas carrier, whatever its origin. The concentration *C* of radon transported by a gas carrier at a velocity *v* along the z direction is described by the following transport equation:


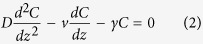


where *D* is the diffusion coefficient and γ the radon decay constant (2.1 × 10^−6^ s^−1^). The three contributions in Eq. 1 refer to the diffusion, advection and radioactive decay processes, respectively. Assuming a concentration *C*_*0*_ at a depth z = z_0_, the solution of the transport equation (Equation 1) provides an estimate of the concentration *C* at the sub-surface^44^,^48^:


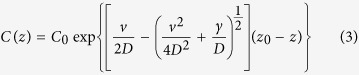


Therefore, the ratio *C/C*_*0*_ at the ground surface z = 0 is given by:


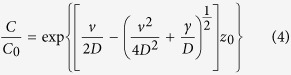


Using an average value of the diffusion coefficient in the soil D = 5 × 10^−2^ cm^2^ s^−1^, the *C/C*_*0*_ ratios are explored in the parameter space (z_0_, v).

The computed ratios *C/C*_*0*_ have been compared with the temporal changes of radon signal measured at ground surface. ERN4 probe measured significant fluctuations in radon activity (generally from ~300 Bq/m^3^ to >7000 Bq/m^3^, represented as daily average values). In particular, the highest radon values ranged from ~5000 Bq/m^3^ (reached on late March 2010, i.e., after ~7 weeks after the first anomaly) to ~7000 Bq/m^3^ (during the second and third anomalies, on January - February 2011).

Considering the average radon concentration of the shallowest volcanic rocks (12,814 Bq), the deeper volcanic rocks (79,044 Bq) and the sedimentary basement (57,706 Bq), the observed ratio *C/C*_*0*_ ranges from 0.004 to 0.121 (see the bottom of Fig. 6). These ratios could provide constraints on the gas carrier velocity.

If we assume that the source of radon activity is located at a depth between 0 and 600 m (i.e., the shallower volcanic sequence), radon values of 300 Bq/m^3^ detected at surface by ERN4 probe implies a gas carrier velocity in the range 5–28 m/day (see the pink box in the left lower corner in Fig. 6). For example, the velocity of 28 m/day is obtained considering the intersection between the vertical line corresponding to 600 m (z_0_), the ratio *C/C*_*0*_ = 0.023 (see Table in Fig. 6 for 300 Bq/m^3^ in average radon activity), and reading the correspondent value of velocity on y-axis. If radon values increase up to 5000–7000 Bq/m^3^, the ratio *C/C*_*0*_ ranges from 0.39 to 0.546 and consequently the gas carrier velocity increases up to unrealistic high values (>>100 m/day). This means that the shallowest portion of the volcanic succession (0–600 m) cannot be the source of the highest values of radon (5000–7000 Bq/m^3^) recorded by ERN4 probe. Hence, this source must be deeper than 600 m.

If we assume that radon was issued from deeper volcanic sequence (600–800 m), i.e. the rocks with higher content in U, radon values of 300 Bq/m^3^ would imply a gas carrier velocity of 20–27 m/day. In the case of the highest radon values (up to 5000–7000 Bq/m^3^) the ratio *C/C*_*0*_ would range from 0.063 to 0.089, therefore the gas carrier velocity would increase up to 27–53 m/day and 53–59 m/day, respectively.

Finally, if radon was issued from the sedimentary basement (>800 m), radon values of 300 Bq/m^3^ would be compatible with gas carrier ascent velocities of 28–38 m/day, of 38–41 m/day and of 41–58 m/day, at depths of 800–1100 m, 1100–1200 and 1200–1400 m, respectively. The highest radon values recorded at the surface by ERN4 suggest a high velocity of the gas carrier; in case of Rn = 5000 Bq/m^3^, the velocity is 38–81 m/day (if radon came from 800–1100 m deep), 81–89 m/day (depth of 1100–1200 m), or >100 m/day (depth >1200 m). Lastly, in case of Rn = 7000 Bq/m^3^, the ascent rate is 93 m/day (if radon came from 800–1100 m deep), and >93 m/day in case of greater depths of the source.

These results lead to some considerations:

1. Low values of radon activity (300 Bq/m^3^) detected at the topographic surface have probably been released by the rocks belonging to the shallow stratigraphic sequence (0–800 m) and ascended quite slowly (1–27 m/day).
2. The highest radon values, recorded following the first major anomaly (up to 5000 Bq/m^3^) indicate a source located in the transition zone volcanic rocks-sedimentary basement (600–1200 m), with ascending velocity of 53–88 m/day.
3. The highest radon values, recorded during the second and third major anomalies (up to 7000 Bq/m^3^), may be produced by a source in the same transition zone (600–1200 m), but with higher gas carrier velocity (60–94 m/day).
4. Surface values of radon >7000 Bq/m^3^ require gas carrier velocity >94 m/day, probably with the contribution of a source located also in deeper rocks (1200–1400 m).

## Discussion

Several conditions may affect soil-radon activity as measured in this study: 1) the concentration of parent radionuclides in the different layers of rock in the sub-soil; 2) the surface to volume ratio of the soil and sub-soil clasts (a higher surface area to volume ratio will lead to a higher efficiency in the escape of radon from the rock matrix); 3) average bulk permeability of the sub-surface rocks, together with the type of permeability (primary or secondary); 4) changes in the advection-driven transport due to variations in the deep gas flux.

The variations in radon activity recorded at Etna by ERN4 probe could be ascribed to changes in the gas carrier velocity, whose flux in the sub-surface may be influenced by temporal variations in the rock properties, because of seismic and/or volcanic activity.

The intense seismicity recorded during the study-period could have induced rock fracturing and opening of new voids, thus enhancing the porosity and permeability that promote gas release. Fault-zone porosity and permeability are expected to change both before and during seismic processes due to a number of factors, namely the formation of new cracks, changes from ineffective (or isolated) to effective (or connected) porosity (rearrangement of the interconnection chains between existing voids), grain size comminution and gouge evolution^49^.

**Figure 7:**
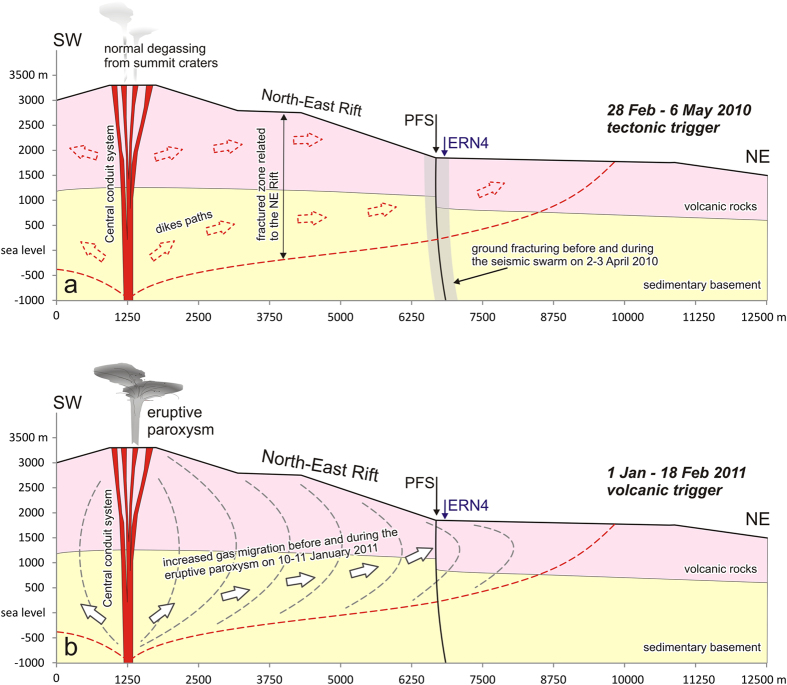
Schematic geological sections of the summit and NE Rift of Mt. Etna volcano, and theoretical models to explain the radon anomalies recorded by ERN4 probe. (**a**) Tectonic trigger: the progressive accumulation of tectonic stress produced the gradual micro-fracturing of the rocks along the fault plane (PFS, light grey), thus changing their porosity and permeability; this process gradually increased the velocity of gas carrier starting from ~7 weeks before the seismic swarm of April 1–2, 2010. (**b**) Volcanic trigger: the increase and pulses of the magmatic gas before/during the onset of the 2011 volcanic paroxysms (white arrows and dashed grey lines) caused a sharp increase of the radon values during the second and third periods of anomaly.

The first anomaly of radon values recorded in the Piano Provenzana area by ERN4 probe and the following values was characterized by a drop followed by a gradual, steady increase in radon activity that reached its highest values (daily average of ~5000 Bq/m^3^) on March 29, i.e., three-four days before the seismic swarm of April 1–2. The portion of the fault involved in the swarm was very shallow (<1–2 km) and the most powerful earthquakes occurred at <1 km depth; noteworthy, the highest radon values recorded during the first anomaly, were associated with a source modeled at comparable depth (600–1200 m). Therefore, we suppose that the trigger of this radon anomaly can be identified in the progressive accumulation of tectonic stress on the fault plane, with consequent gradual micro-fracturing of the rocks, thus changing their porosity and permeability. This mechanism produced at its onset the negative radon anomaly because of dilution of this gas following increased atmospheric circulation in the soil. Later on, this process led to a gradual increase in the velocity of gas release at the sub-surface as early as ~7 weeks before the seismic swarm. This interpretation is summarized in Fig. 7a. Noteworthy, similar patterns in radon emissions before some notable earthquakes were observed worldwide, e.g., Tangshan^50^ in 1976 and Kobe^51^ in 1995.

The two anomalies recorded in 2011 differ from the first one in terms of maximum radon values (7000 Bq/m^3^), and of marked oscillation of radon values both before and after reaching the peak. Due to the temporal link with the onset of several paroxysmal eruptions at Mt. Etna, these anomalies were likely not triggered by a tectonic process, but possibly by a volcanic event. The 12–13 January 2011 paroxysmal eruption is, in fact, a key moment in the recent history of Mt. Etna, because starting from that event the New Southeast Crater began to grow^31^,^32^,^33^,^34^. These two anomalies occurred during the first two paroxysmal eruptions of the New Southeast Crater, i.e., 4 days after the first volcanic paroxysm and 9 days before the second one. Similar to what was observed in the period between the first anomaly and the occurrence of the seismic swarm of 2010, radon emissions in 2011 were also characterized by a steady increase during the weeks before the first paroxysm, but differently from the pre-seismic increase, in this case the increasing trend was characterized by larger oscillations of radon values.

Taking into account the geometry of the volcano’s central conduit and the supposed depth of the NE Rift, structurally linked to the summit conduits^25^,^42^, we drew a conceptual model to explain the increase and lateral expansion of the magmatic gas before and during the beginning of the 2011 volcanic paroxysms (white arrows in Fig. 7b). Due to the location of the ERN4 probe, i.e., very close to the NE Rift, we presume that pulses of magmatic gas may have caused the variations of the radon values during early 2011. A similar pulsating behavior in soil radon emissions was already observed in the data from a probe located near the summit cone of Mt. Etna before paroxysmal episodes occurred at the Southeast Crater in 1998. This was explained by a possible increase in magmatic gas pressure inside the shallow conduit of the volcano^9^.

Summarizing, the ERN4 radon probe installed at Piano Provenzana (1800 m asl) on the NE flank of Mt. Etna volcano recorded important data during recent tectonic and volcanic events. These data allowed us to better understand how and why soil radon varies on an active volcano. We analyzed the soil radon activity, the main meteorological parameters and several soil samples to measure their uranium content. The main results are the following:

1. In the NE sector of Mt. Etna, volcanic rocks older than ~90–100 ky are richer in ^238^U than those younger.
2. The radon recorded by ERN4 probe comes mainly from these older volcanic rocks and from the shallowest portion of the sedimentary basement of the volcano.
3. The source of radon that produced the highest activity values (daily averages in the range 5000–7000 Bq/m^3^) is in any case somewhat shallow, probably <1400 m.
4. These radon values can be justified by considering a gas carrier velocity of >50 m/day.

Three periods of significant radon anomaly, based on linear regression analysis on radon data in order to filter out all relevant external influences, were detected (Fig. 4). The first radon anomaly (February 2010) had a tectonic trigger. It was characterized by negative values followed, during the next 7 weeks, by a steady and regular increase in radon activity. The highest values were reached immediately before a seismic swarm affecting the Pernicana Fault System on 2–3 April 2010.

The second period of radon anomaly (January and February 2011) likely had a volcanic trigger. These two anomalies occurred during the first two paroxysmal eruptions of the New South-East Crater. Radon emissions in 2011 were also characterized by a steady increase during the weeks before the first paroxysm, but differently from the pre-seismic increase, in this case the increasing trend was characterized by larger oscillations of radon values caused by pulses of magmatic gas coming from the central conduit of Mt. Etna and advectively traveling through the NE Rift.

We believe that the results of the present investigation mark a significant first step towards better understanding the endogenous mechanisms that cause changes in soil radon migration on active volcanoes like Mt. Etna. Some light is shed on the complex interplay between tectonic activity, volcanic eruptions and gas release through faults. Our approach could be used in other volcanoes throughout the world to test the validity of our analysis.

## Methods

From 19 November 2009 to 13 April 2011, the ERN4 probe recorded 48,994 values of radon activity (expressed in Bq/m^3^), down-hole temperature (in °C) and atmospheric pressure (in mbar) every 15 minutes. In addition, we daily recorded data of air temperature, thickness of the snow cover and rainfall, measured in the same area by the weather stations managed by Meteomont (http://www.meteoam.it/?q=bollettini/meteomont) and by Meteo Sicilia (http://www.meteosicilia.it) (Fig. 1). When radon activity fell below 178 Bq/m^3^, namely the instrumental lower limit, the Barasol probe gave a null value. The highest value of radon was 23,384 Bq/m^3^ and was measured on 12 April 2011. In order to make them comparable, we calculated the daily averages of each parameter measured by the ERN4 probe (Fig. 2).

Supplementary Tables S1–4 show the statistics for the acquired parameters and Fig. 2 shows their daily averages during the studied time interval, including the radon values predicted by the linear regression model according to the procedure described above (see Results).

In order to evaluate the seismic activity affecting the area (Lat 37.76°N-37.83°N, Lon 14.95°E-15.21°E) during the analyzed period, we studied 116 earthquakes that occurred from 2 November 2009 to 13 April 2011, examining the database of INGV-OE in Catania (http://www.ct.ingv.it/it/terremoti-recenti-etna/catalogo-strumentale.html).

Petrographic characterization and bulk-rock geochemistry analyses have been carried out on ten collected rock samples; the latter aimed at determining the concentration of trace elements with special regards to U and Th. The analytical results, the method used to analyze the rock samples and information about the location of the rock sampling points are shown in Supplementary Table S5 and Supplementary Map S6.

## Additional Information

**How to cite this article**: Neri, M. *et al.* Soil radon measurements as a potential tracer of tectonic and volcanic activity. *Sci. Rep.* **6**, 24581; doi: 10.1038/srep24581 (2016).

## Supplementary Material

Supplementary Information

## Figures and Tables

**Table 1:** Location and stratigraphic position of the collected rock samples, listed from the youngest and shallowest (top) to the oldest and deepest (bottom).

#	Locality	lat (°)	lon (°)	Unit /Formation	Age	litothypes	^238^U (Bq)	
R8	Piano Provenzana	37.799303	15.040696	Pietracannone Formation, upper member, Monte Ponte di Ferro	122 BC–3.939 y	lava	18603	Mt. Etna volcano
R3	Clan dei Ragazzi	37.806560	15.066674	Pietracannone Formation lower member, Pineta di Linguaglossa	3.939 y–15 ky	lava	8735	
R4	Ripe della Naca	37.772032	15.106085	Pietracannone Formation lower member, Casa S. Buco	3.939 y–15 ky	lava	5700	
R2	Due Monti	37.788706	15.061662	Portella Giumenta Formation, Ragabo member	15.05 ky	lava	18220	
R1	Monte Scorsone	37.741769	15.077817	Monte Scorsone Formation	99.9–101.8 ky	lava	67608	
R7	Ripe della Naca- hornitos zone	37.775670	15.102090	Timpa Formation, S. Maria La Scala member	129.9–154.9 ky	lava	90479	
R5	Linguaglossa-Castiglione road	37.872063	15.119493	Flysh of Capo d’Orlando	Upper Oligocene–Lower Burdigalian	silt	82528	Sedimentary Basement
R6	Linguaglossa-Castiglione road	37.869810	15.120810	Flysh of Capo d’Orlando	Upper Oligocene–Lower Burdigalian	arkose sandstone	55532	
R9	Piedimonte Etneo	37.812721	15.184126	Flisch of Piedimonte	55.8–33.,9My	sandstone	53757	
R10	Piedimonte Etneo	37.812721	15.184126	Monte Soro Unit	99.6–65.5My	marl	39008	

The content of ^238^U was calculated on the basis of the chemical analyses shown in Supplementary Table S5. The location of the rock sampling sites is shown in Supplementary Map S6. The age of sampled rocks is from Branca *et al.*^27^.
