# Supplementary material for: Soil radon measurements as a potential tracer of tectonic and volcanic activity
Source: Sci Rep. 2016 Apr 15;6:24581. doi: 10.1038/srep24581 (PMC4832328; doi:10.1038/srep24581)
Supplement: Supplementary Information [file srep24581-s1.pdf]

Soil radon measurements as a potential tracer of tectonic and volcanic activity

Marco Neri [(1)]*, Elisabetta Ferrera [(2)], Salvatore Giammanco [(1)], Gilda Currenti [(1)], Rosolino Cirrincione [(2)], Giuseppe Patanè [(2)], Vittorio Zanon [(3,4)]

(1) Istituto Nazionale di Geofisica e Vulcanologia, Osservatorio Etneo, Sezione di Catania, Piazza Roma, 2 - 95123 Catania
(2) Dipartimento di Scienze Biologiche, Geologiche e Ambientali – Università di Catania. C.so Italia 57, 95129 Catania
(3) Centro de Vulcanologia e Avaliação de Riscos Geológicos, Universidade dos Açores, Ponta Delgada, Portugal
(4) Institut de Physique du Globe de Paris, 1, rue Jussieu - 75238 Paris cedex 05, France

* *Corresponding author*: marco.neri@ingv.it

Supplementary Information

Supplementary Table S1

	min	*max*	*mean*	*standard deviation*	*mode*	*median*
**Rn** (Bq/m^3)*	0	23384	3300	2159	0	3493
**T soil** (°C)*	4.4	19.8	8.9	2.6	12.7	9.4
**T air** (°C)	-6.8	23.7	6	6.6	0	5.2
**P** (mbar)*	794	834.7	818.2	6.3	823.1	819.1
**snowfall** (cm)	0	138	16	34	0	0
**rainfall** (mm)	0	99.8	3.2	10.4	0	0

Basic statistics for the parameters acquired daily by the ERN4 probe (*) and by Meteomont and Meteo Sicilia weather stations. Rn = radon activity; T soil = temperature at 220 cm below the ground surface, inside the PVC pipe; P = atmospheric pressure; T = air temperature at 100 cm above the ground surface.

Supplementary Table S2

	**radon**	**sensor temp.**	**air press.**	**snow**	**air temp.**	**rain**
**radon**	1.000	-0.145	-0.156	0.305	-0.421	0.036
**sensor temp.**		1.000	0.029	0.191	0.073	-0.050
**air press.**			1.000	-0.201	0.360	-0.161
**snow**				1.000	-0.444	-0.067
**air temp.**					1.000	-0.046
**rain**						1.000

Correlation matrix among the daily averages of measured parameters during the study period.

Supplementary Table S3

Parameters	Mean group 1 (n = 511)	Mean group 2 (n = 511)	Wilcoxon test T	Z	p-level
sensor T° vs. air T°	8.900521	6.9995	39126.00	5.19	2.11E-07
snow vs. sensor T°	16.02544	8.9005	46238.00	5.74	9.45E-09
snow vs. air T°	16.02544	6.9995	41679.00	4.31	1.64E-05
air press vs. sensor T°	818.2257	8.90052	0.00	19.59	0.00
air press vs. snow	818.2257	16.02544	0.00	19.59	0.00
air press vs. air T°	818.2257	6.99946	0.00	18.68	0.00
sensor T° vs. rain	8.9005	2.636986	12800.00	15.75	0.00
air press vs. rain	818.2257	2.636986	0.00	19.59	0.00
snow vs. rain	16.0254	2.636986	0.00	19.59	0.00
air T° vs. rain	6.9995	2.636986	9163.50	6.07	1.28E-09

Results of the Wilcoxon[39] test performed on the meteorological parameters acquired, calculated on pairs of groups. The null hypothesis (i.e., the mean of group 1 is statistically the same as that of group 2) could be rejected in all cases at the 5% (α = 0.05) significance level, because the calculated probabilities (p-level) for each pair of parameters are much lower than the critical limit in the case of a valid null hypothesis. T is the summation of the ranks; Z is the standardized normal distribution.

Supplementary Table S4

No. of variables	Variables in the regression equation	R^2
1	Rn = *f*(Ta)	0.2038
2	Rn = *f*(Ta, Pa)	0.2181
3	Rn = *f*(Ta, Pa, Tp)	0.2186
4	Rn = *f*(Ta, Pa, Tp, S)	0.2187
5	Rn = *f*(Ta, Pa, Tp, S, R)	0.2187

Best subset search process for the variables acquired. Rn = radon activity; Ta = air temperature; Pa = barometric pressure; Ts = probe temperature; S = snowfall; R = rain fall.

Supplementary Table S5

ppm	R1	R2	R3	R4	R5	R6	R7	R8	R9	R10
^{85}Rb	51.53	62.12	46.89	24.78	129.57	141.64	39.16	34.54	81.81	71.02
^{88}Sr	1287.19	1135.97	1414.48	929.54	136.99	178.94	1348.28	1328.01	120.28	863.6
^{89}Y	24.8	33.99	27.42	23.53	23.88	20.59	25.48	25.17	9.74	11.84
^{90}Zr	264	329.52	266.65	207.07	82.54	22.21	263.77	271.84	38.22	33.66
^{93}Nb	114.35	118.36	93.33	70.17	20.1	11.27	89.38	80.92	11.64	7.3
^{139}La	76.69	107.19	86.54	63.62	62.54	55.43	86.59	82.9	20.09	16.29
^{140}Ce	144.35	187.72	159.89	106.25	110.5	94.02	163.69	151.55	39.88	27.73
^{141}Pr	16.16	22.8	18.86	13.17	13.6	11.79	18.76	17.3	4.63	3.38
^{146}Nd	62.01	81.82	69.67	51.61	51.77	43.99	68.82	66.06	18	12.37
^{147}Sm	9.92	12.85	11.21	9.13	9.75	8.23	10.95	10.75	3.57	2.53
^{153}Eu	2.96	3.65	3.26	2.71	1.79	1.99	3.16	3.11	0.81	0.56
^{157}Gd	9.19	11.72	10.12	8.32	8.36	7.24	9.84	9.54	3.13	2.37
^{159}Tb	1.22	1.52	1.32	1.18	1.17	1.05	1.27	1.25	0.47	0.37
^{163}Dy	5	6.29	5.38	5.1	4.79	4.28	5.14	5.11	2.05	1.82
^{165}Ho	0.94	1.19	1.01	0.96	0.89	0.76	0.96	0.97	0.39	0.38
^{166}Er	2.45	3.21	2.69	2.46	2.37	1.88	2.55	2.52	1.05	1.05
^{169}Tm	0.37	0.5	0.41	0.36	0.38	0.28	0.38	0.38	0.17	0.18
^{172}Yb	2.1	2.88	2.34	2.03	2.27	1.56	2.2	2.18	1	0.99
^{175}Lu	0.31	0.44	0.35	0.3	0.35	0.23	0.33	0.33	0.15	0.15
^{178}Hf	5.3	6.63	5.38	4.26	2.3	0.68	5.34	4.98	1.05	0.78
^{181}Ta	3.91	4.09	3.31	2.45	1.24	0.73	3.18	2.91	0.69	0.4
^{232}Th	14.06	23.08	17.07	9.2	18.13	14.98	16.81	15.94	6.7	3.76
^{238}U	4.68	6.84	5.28	3.44	3.35	2.26	5.21	5.12	2.18	1.58

Results of the chemical analysis on the rock samples collected on Mt. Etna to determine trace elements. Analyses were carried out by inductively coupled plasma mass spectrometry (ICP-MS). All values are expressed in ppm.

Rock samples were analyzed for trace elements composition by inductively coupled plasma mass spectrometry (ICP-MS) at the Department of Physics and Earth Science of the University of Ferrara. Between 0.1 and 0.2 g of finely pulverized sample reacted with 6 ml HF (40% Merck Suprapur) and 3 ml of HNO_3 (65% Merck Suprapur) in an ultrasonic bath for 10 minutes and then left to stand overnight. The ultrasound bath was repeated again before heating at 170 °C on a hot plate. Once 3 ml of HF and with 3 ml of HNO_3 have been then added to the dry sample, and then left to stand overnight. Ultrasound bath and heating is then repeated, before adding 4 ml of HNO_3 and drying again. Finally, 2 ml of HNO_3 were added and the sample before pouring it into a volumetric flask. At this point, samples were spiked with internal multi-elementary standards of Re, Rh, In and Bi at a concentration of 10 ppb. The volumetric flask was then filled up to the notch with 100 ml of MilliQ water.

Supplementary Map S6

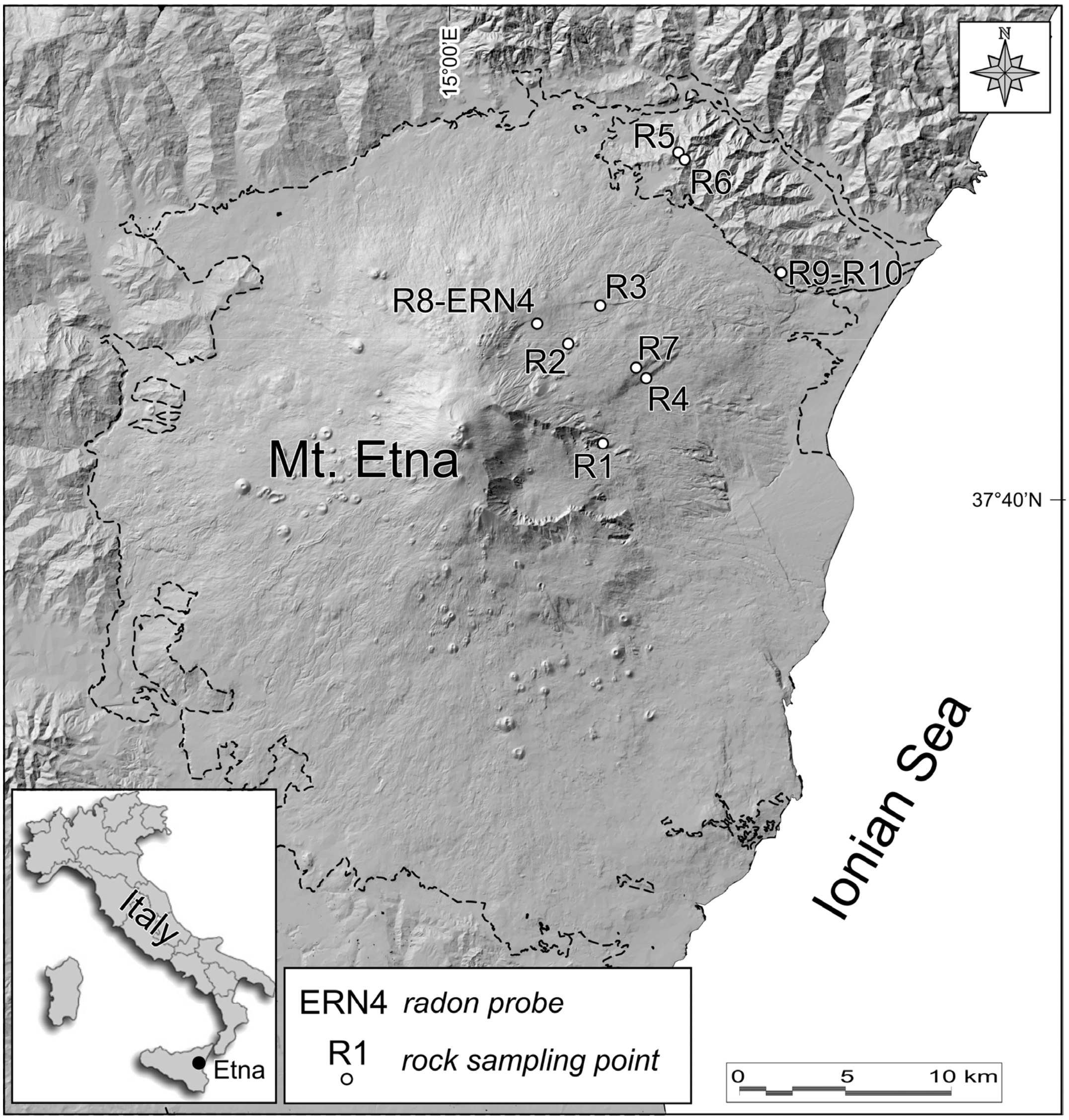

Location Map of the rocks sampling points (R1-10) and of the radon probe (ERN4). The dotted lines show the limits of Mt. Etna's volcanics. This figure and the map of Italy were generated using CorelDRAW graphic suite X4 software (http://www.corel.com/it/). The main map was generated using a DEM owned by INGV.
